# Resolvin D1 mitigates energy metabolism disorder after ischemia–reperfusion of the rat lung

**DOI:** 10.1186/s12967-016-0835-7

**Published:** 2016-03-24

**Authors:** Qifeng Zhao, Ji Wu, Qingwang Hua, Zhiyong Lin, Leping Ye, Weixi Zhang, Guowei Wu, Jie Du, Jie Xia, Maoping Chu, Xingti Hu

**Affiliations:** The Department of Children’s Cardiovascular and Thoracic Surgery, Children’s Heart Center, the Second Affiliated Hospital, Yuying Children’s Hospital, Institute of Cardiovascular Development and Translational Medicine, Wenzhou Medical University, 325000 Wenzhou, People’s Republic of China; The Department of Children’s Respiration Medicine, The Second Affiliated Hospital and Yuying Children’s Hospital, Wenzhou Medical University, 325000 Wenzhou, People’s Republic of China; The Department of Children’s Cardiovascular Medicine, Children’s Heart Center, the Second Affiliated Hospital, Yuying Children’s Hospital, Institute of Cardiovascular Development and Translational Medicine, Wenzhou Medical University, 325000 Wenzhou, People’s Republic of China; Wuhan Medical & Healthcare Center for Woman and Children, 430015 Wuhan, People’s Republic of China

**Keywords:** Resolvin, Lung ischemia/reperfusion injury, Inflammatory factor, Oxidative stress, Energy metabolism

## Abstract

**Background:**

Energy metabolism disorder is a critical process in lung ischemia–reperfusion injury (LIRI). This study was aimed to determine the effects of resolvin D1 (RvD1) on the energy metabolism in LIRI.

**Methods:**

Forty Sprague–Dawley rats were divided into the following groups: Sham group; untreated ischemia–reperfusion (IR) control; IR treated with normal saline (IR-NS); and IR treated with RvD1 (IR-RV) (100 μg/kg, iv). LIRI and energy metabolism disorder were determined in these rats.

**Results:**

The results revealed that the levels of interleukin (IL)-1β, tumor necrosis factor-α, IL-10, monocyte chemoattractant protein-1, macrophage inflammatory protein-2, cytokine-induced neutrophil chemoattractant-1, injured alveoli rate, apoptosis index, pulmonary permeability index, malondialdehyde, ADP, and lactic acid were increased, whereas the levels of ATP, ATP/ADP, glycogen, Na^+^–K^+^-ATPase, superoxide dismutase, glutathione peroxidase activity, pulmonary surfactant associated protein-A, and oxygenation index were decreased in rats with LIRI. Except for IL-10, all these biomarkers of LIRI and its related energy metabolism disorder were significantly inhibited by RvD1 treatment. In addition, histological analysis via hematoxylin–eosin staining, and transmission electron microscopy confirmed that IR-induced structure damages of lung tissues were reduced by RvD1.

**Conclusion:**

RvD1 improves the energy metabolism of LIRI disturbance, protects the mitochondrial structure and function, increases the ATP, glycogen content and Na^+^–K^+^-ATPase activity of lung tissue, balances the ratio of ATP/ADP and finally decreases the rate of apoptosis, resulting in the protection of IR-induced lung injury. The improved energy metabolism after LIRI may be related to the reduced inflammatory response, the balance of the oxidative/antioxidant and the pro-inflammatory/anti-inflammatory systems in rats.

**Electronic supplementary material:**

The online version of this article (doi:10.1186/s12967-016-0835-7) contains supplementary material, which is available to authorized users.

## Background

Lung ischemia–reperfusion injury (LIRI) occurs in many cases, such as the cardiopulmonary bypass, lung transplantation and post enucleation of pulmonary embolism [1–3]. In addition, LIRI is also involved in other situations including shock, respiratory failure caused by lower limb and trunk ischemia–reperfusion (IR) and acute respiratory distress syndrome [4–6]. Recently, much attention has been paid to the pulmonary dysfunction resulted from LIRI. However, due to the complex of the mechanism of LIRI and its involved factors, the effective methods for prevention and treatment of LIRI are still very limited. More recently, the energy metabolism disorder has been found to be the key process of ischemia–reperfusion injury (IRI) [7]. Studies have demonstrated that the protection of the energy status and the amelioration of metabolic disorders could remarkably reduce the organ IRI [8, 9]. Nevertheless, the energy metabolism of LIRI has its own characteristics, and there has been little research in this new area.

The endogenous lipid mediators, such as resolvin (Rv) and lipoxin, have been confirmed to have the anti-inflammatory effect in many studies [10, 11]. These specialized pro-resolving mediators have conserved structures with many functions in host defense, pain, organ protection and tissue remodeling [12]. In addition, these bioactive substances have been found to have a protective effect on organ ischemia reperfusion injury [13–19]. Recently, it has been reported that RvD1 could preserve the activity of Na^+^–K^+^-ATPase and relieve the lung injury induced by oxidative stress and inflammatory response [20, 21]; however, little research has been reported about the effect of RvD1 on the energy metabolism of LIRI.

In this study, we aim to investigate the protective effect and the related mechanisms of RvD1 on the lung energy metabolism caused by LIRI in rats, and hope to provide a new idea and its experimental evidence for the treatment of LIRI. In particularly, through the intravenous injection of RvD1, we studied the effects of RvD1 on the ATP, ADP, glycogen, lactic acid content, the activity of Na^+^–K^+^-ATPase, the inflammatory response and the oxidative stress in lung tissue. Meanwhile, the pathological changes, apoptosis rate and pulmonary function in the lung tissue were also evaluated.

## Methods

### Rat model of LIRI

The animal procedures were approved by Wenzhou Medical University Animal Care and Use Committee, which were certified by the Chinese Association of Accreditation of Laboratory Animal Care and were consistent with the Guide for the Care and Use of Laboratory Animals [updated (2011) version of the NIH guidelines]. Male Sprague–Dawley (SD) rats (8 weeks old) were fed a standard diet and maintained in the controlled environment of the animal center at 25 ± 1 °C under a 12 h light–dark cycle. The LIRI rat model was induced by the following procedures. Briefly, rats were anesthetized by an intraperitoneal injection of 10 % chloral hydrate (300 mg/kg body weight) and placed in a supine position. The animals were then intubated for artificial ventilation with oxygen using a small animal breathing machine (tidal volume 5 ml, frequency 70 per min) and electrocardiograph monitor. Thoracotomy was performed at the anterior lateral side of the left fourth intercostal. The muscular layer and pleura were gentle dissected to expose the heart and lung. After that, the hilum of left lung was dissociated and the artery clamp was used to pass through the hilum of lung from the upper right to lower left. The whole clamped left hilum was clearly exposed by slightly stirring up the clamp. Before blocking, heparin was injected by i.v. (1 mg/kg body weight). After ischemia for 45 min, the artery clamp was removed (no blocking in sham group) and then reperfusion was started and lasted for 150 min. During the reperfusion time, 0.5 ml normal saline (NS) was injected by i.v. every hour to maintain the body fluid. Finally, after the chest wall was closed and the body temperature was maintained using a 37 °C warming plate.

### Animal grouping and treatments

Resolvin D1 (RvD1, C_22_H_32_O_5_, 7S,8R,17S-trihydroxy-4Z,9E,11E,13Z,15E,19Z-docosahexaenoic acid, please see Additional file 1: Figure S1 in the Supplementary Material) was purchased from Cayman Chemical Company (Ann Arbor, USA). Forty SD rats were randomly divided into four groups (10 rats/group) as follows: (1) Sham group: no blocking of hilum of left lung after thoracotomy; (2) untreated IR control (IR-C) group: blocking of hilum of left lung after thoracotomy for 45 min, followed by reperfusion for 150 min; (3) IR treated with normal saline (IR-NS) group: after blocking the hilum of left lung for 45 min, reperfusion for 10 min then injection 2 ml/kg NS by formal vein, and the total reperfusion time was 150 min; (4) IR treated with RvD1 (IR-RV) group: after blocking the hilum of left lung for 45 min, reperfusion for 10 min then injection 100 μg/kg RvD1 by formal vein and the total reperfusion time was 150 min.

### Blood and tissue harvest

Blood samples were collected in each group immediately before thoracotomy (T_1_) and after the experiments (T_2_). In Sham group, T_2_ was achieved after 195 min of the artery clip across the left hilus pulmonis. For all the other groups, T_2_ blood samples were obtained after 150 min of reperfusion. Rats were killed after blood collection. The bronchoalveolar lavage fluid (BALF) was then collected by washing the airways of the left lungs three times with a total of 5 mL of phosphate buffer solution (PBS) through a tracheal cannula (recovery rate >80 %), which was pooled and centrifuged at 3000 rpm/min for 15 min for further use. At time point of T_2_, the left lung tissue of rats was dissected to measure the W/D value (wet to dry weight ratio, W/D). Other lung tissue were fixed in 4 % paraformaldehyde or frozen in −70 °C refrigerator for further analysis.

### Lung tissue hematoxylin–eosin (HE) staining and transmission electron microscopy (TEM)

The obtained lung tissue samples at T_2_ were fixed in 10 % neutral-buffered formalin and subsequently embedded in paraffin. Tissue sections (5 μm thick) were stained with HE using a standard protocol and analyzed by light microscopy.

For TEM examination, lung tissue containing a 2 mm portion from the edge of the incision were immediately fixed in 0.1 M phosphate buffer containing 2.5 % glutaraldehyde and 2 % paraformaldehyde for at least 4 h. Samples were made of resin-embedded blocks, which were cut into 60 ~ 80 nm ultrathin sections with an ultra-microtome (PT-XL, RMC, USA). The ultrathin sections were placed on carbon coated nickel grids and examined with an H-7500 transmission electron microscope (H-7500, Tokyo, Japan) operating at 80 kV.

### Measurement of injured alveoli rate (IAR)

The IAR, a quantitative evaluation index of lung injury, were obtained in this study. The lung tissue sections stained with HE were observed under the light microscope at 200× visual field. Total 200 alveoli were counted and those containing more than two red blood cells and white blood cells would be considered as the injured ones. Finally, the IAR was obtained [22].

### Measurement of pulmonary surfactant associated protein-A (SP-A)

The lung tissue homogenate was centrifuged and the supernatant was used to determine the concentration of SP-A by a rat ELISA kit (R&D, USA) according to the manufacturer instructions.

### Oxygenation index

The arterial blood gas analysis was performed at time point T_2_ and the ratio of PaO_2_ to FiO_2_ were then obtained and expressed as oxygenation index (oxygenation index = PaO_2_/FiO_2_).

### Lung tissue W/D

The lung tissue W/D is an indicator of the lung tissue edema. About 1 g of lung tissue were measured and named as wet weight. The tissue was then kept in 70 °C electrothermal constant-temperature dry box for 48 h and the weight of tissue was designed as dry weight. Finally, W/D was calculated and analyzed.

### Pulmonary permeability index (PPI)

Samples of BALF precipitate were centrifuged and the supernate of BALF and blood serum was harvested for total protein analysis using the Bradford method. The ratio of total protein in BALF to the total protein in blood serum was calculated and named as PPI.

### Measurement of Na^+^–K^+^-ATPase activity in the lung tissue

The Na^+^–K^+^-ATPase activity was determined by measuring the release of inorganic phosphate (Pi) from ATP according to the kit protocol (Jiancheng Bioengineering Institute, Nanjing, China). The amount of Pi was measured with the malachite green dye method.

### Measurement of glycogen and lactic acid content in the lung tissue

The lung tissue homogenate was centrifuged and the supernatant was used to determine the concentration of glycogen by a rat ELISA kit (R&D, USA) according to the manufacturer instructions. The lactic acid content was also measured according to the manufacturer instructions (Jiancheng Bioengineering Institute, Nanjing, China).

### The ATP, ADP contents and the ratio of ATP to ADP in the lung tissue

The ATP, ADP contents in the lung tissue were detected by the high performance liquid chromatography (HPLC). The ratio of ATP to ADP was then calculated based on the HPLC results.

### Cytokine levels

For cytokine immunoassay, blood samples were collected by femoral venipuncture at time points T_1_ and T_2_. The serum levels of interleukin (IL)-1β, tumor necrosis factor (TNF)-α and IL-10 were measured using a rat ELISA kit (Boyun Biotech, Shanghai, China) in accordance with the kit instructions.

### Chemokine levels

The lung tissue homogenate was centrifuged and the supernatant was used to determine the concentrations of Monocyte chemoattractant protein (MCP)-1, macrophage inflammatory protein (MIP)-2, and cytokine-induced neutrophil chemoattractant (CINC)-1 by a rat ELISA kit (R&D, Minneapolis, MN, USA) according to the manufacturer instructions.

### Glutathione peroxidase (GSH-PX), superoxide dismutase (SOD) activity and malondialdehyde (MDA) content determination

The lung tissue GSH-PX and SOD activity was determined on frozen tissue using Xanthine Oxidase assay kits (Jiancheng Bioengineering Institute, Nanjing, China). The MDA content was determined on frozen lung tissue by use of the thiobarbituric acid assay kit (Jiancheng Bioengineering Institute, Nanjing, China).

### TdT-mediated dUTP nick end labeling (TUNEL) assay

TUNEL assay was performed to determine the apoptosis of the lung tissue with TUNEL test kit (Roche, USA) according to manufacturer’s instructions. Five fields of view were automatically selected by the Image-Pro Plus version 5.1 image analysis software. The percentage of apoptosis-positive cells was calculated for each field of view. The mean was calculated to obtain the percentage of apoptotic cells, and expressed as apoptotic index (AI). AI = (apoptotic nuclei count/total nucleus count) × 100 %.

### Statistics

All data are presented as mean ± standard error. One-way ANOVA and two-tailed unpaired-samples T tests were used for statistical evaluation of the data. SPSS 17.0 was used for data analysis. A *P* < 0.05 was considered significant.

## Results

### Pathologic and ultrastructure changes of lung tissues

**Fig. 1:**
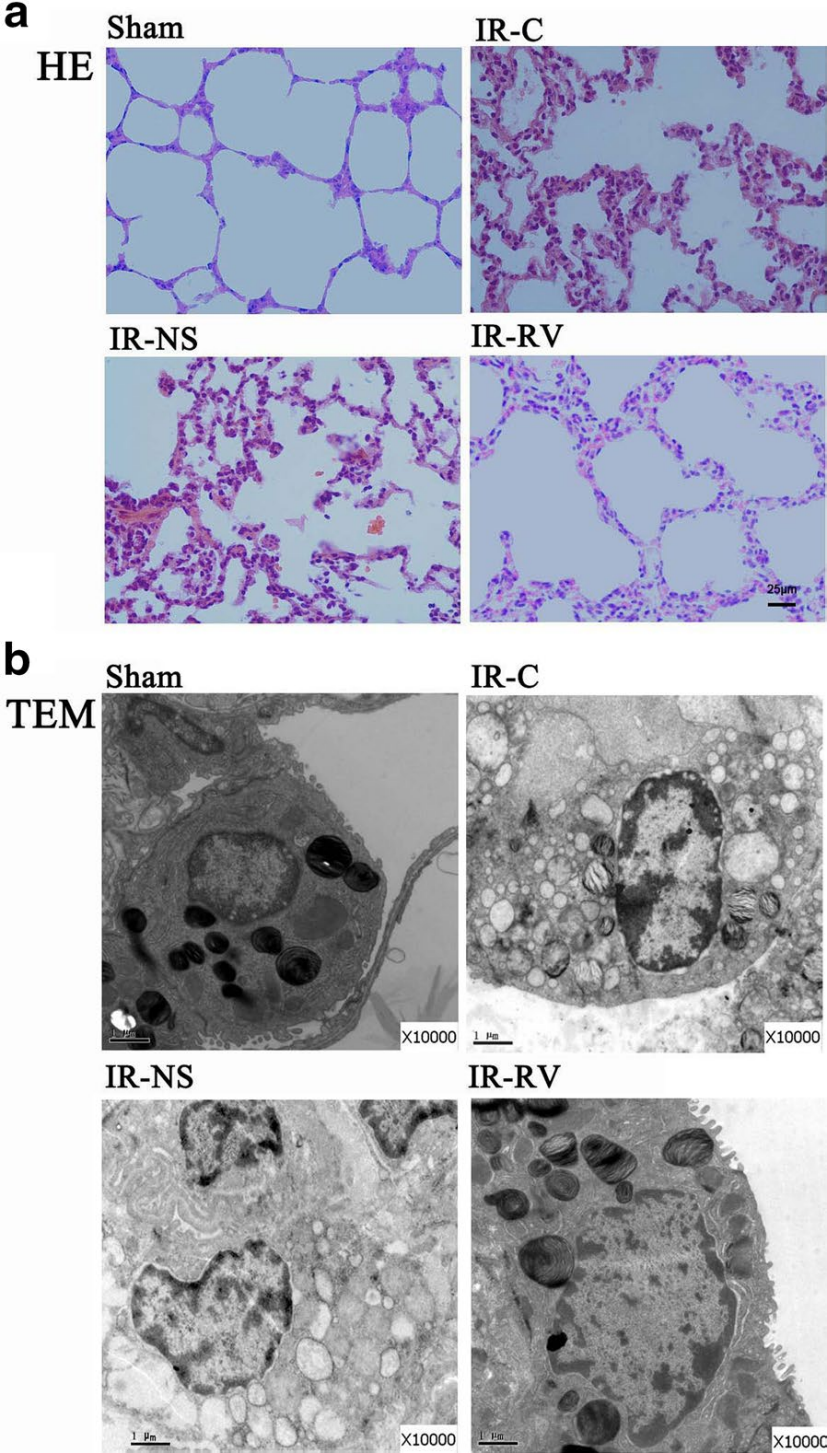
Pathologic changes of lung tissues examined by HE staining and TEM. Lung tissues were dissected and performed with HE staining and TEM analysis. **a** HE staining of the lung tissue sections in the four groups; **b** TEM results of the tissue sections in the four groups. The HE staining results demonstrated no abnormal changes in the sham group; however, pathological changes happened severely in the IR-C and IR-NS group. The histological damages in IR-RV group were reduced compared with the other two IR groups. The TEM results demonstrated the lung tissue ultrastructure was normal with tightly connected pulmonary capillary endothelial cells, intact basement membrane and integral type I and type II alveolar epithelial cells in sham group. Meanwhile, the mitochondrial cristae, microvilli and the lamellar body were clear. However, the ultrastructure was changed in IR-C and IR-NS groups. Their ultrastructure of lung tissue appeared serious abnormalities. In IR-RV group, reduced injuries were found compared to the other two IR groups

The pathological changes of lung tissue determined by HE staining in each group were shown in Fig. 1a. In sham group, the pulmonary alveoli and interstitium were intact with smooth thin alveolar wall and uniform alveolar septal thickness. No abnormality such as the apomorphosis, exudation and neutrophil infiltration was observed. In IR-C group and IR-NS group, damaged alveoli structure, dilated and congestive capillaries were seen. The thickened interstitium was infiltrated with inflammatory cells, while the alveolar lumen was also filled with exudates, red blood cells and neutrophils. The histological condition in IR-RV group was much better than that in IR-NS and IR-C group. Less neutrophil infiltration and only slight dilatation of the capillaries were observed in the IR-RV group.

The ultrastructure of lung tissue was observed by TEM and exhibited in Fig. 1b. In sham group, the lung tissue ultrastructure was normal with tightly connected pulmonary capillary endothelial cells, intact basement membrane and integral type I and type II alveolar epithelial cells. The mitochondrial cristae, microvilli and the lamellar body were clear. However, the ultrastructure was changed in IR-C and IR-NS groups. Their ultrastructure of lung tissue appeared serious abnormalities with swelling pulmonary capillary endothelial cells, mitochondria and shrinking nuclear membrane. Less pinocytosis vesicles could be seen in type I alveolar epithelial cells. Meanwhile, decreased numbers of microvilli and scarce lamellar bodies of the type II alveolar epithelial cells were observed, and a large number of inflammatory cells infiltrated the alveolar septum and capillaries were identified. In IR-RV group, reduced injuries were found compared to IR-C and IR-NS groups. Their pulmonary capillary endothelial cells and mitochondria exhibited slight swelling. More pinocytosis vesicles were seen in type I alveolar epithelial cells; and type II alveolar epithelial cell surface appeared an increasing number of microvilli and lamellar bodies. The alveolar septum showed edema and was still more than normal thickness but had no obvious inflammatory cell infiltration.

### The influence of RvD1 on lung injury and lung function caused by LIRI

**Fig. 2:**
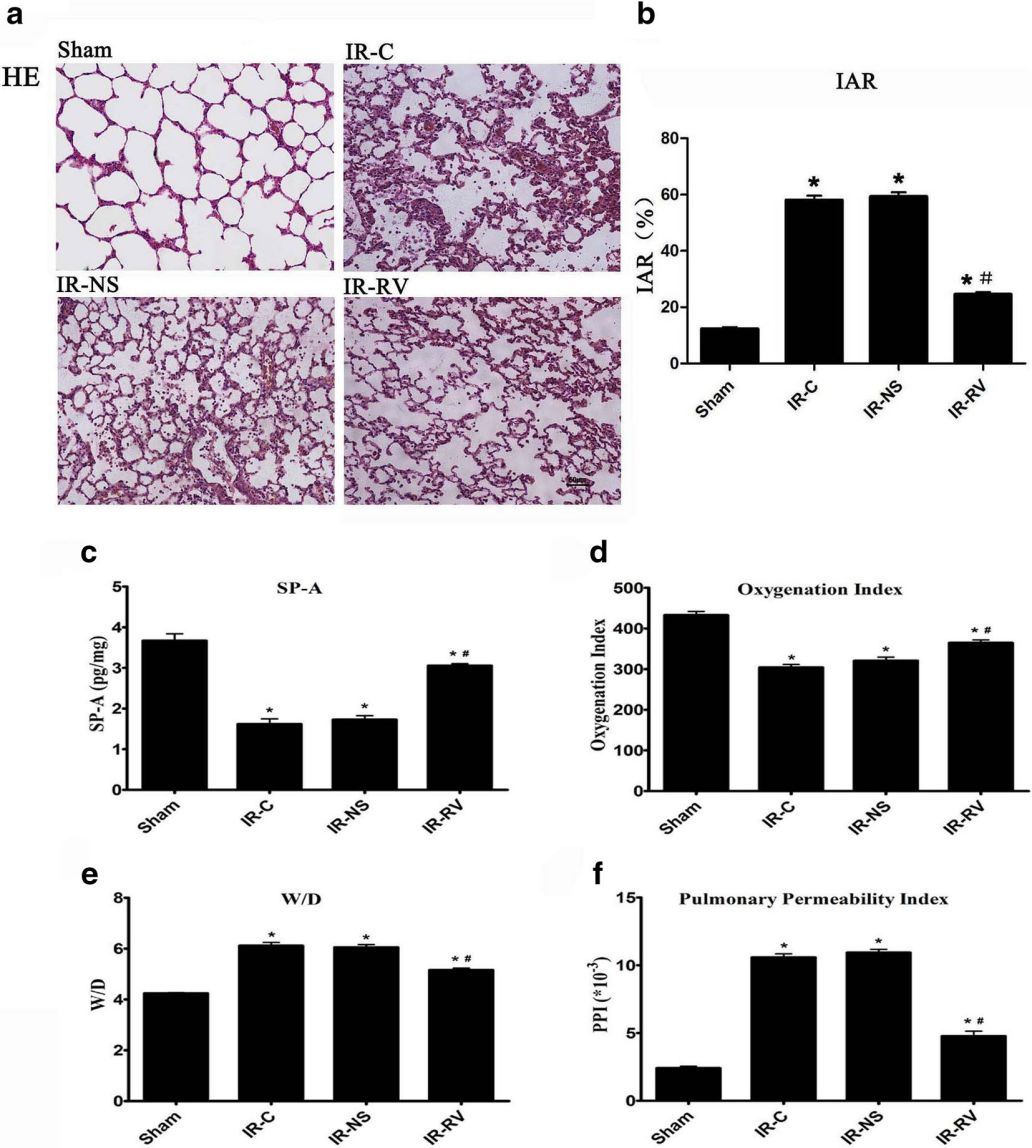
The influence of RvD1 on lung injury and lung function. At time point T_2_, lung tissue, BALF and arterial blood were collected immediately after IR procedure was completed. H&E staining of lung tissues, IAR, SP-A, W/D, oxygenation index and PPI were measured as described in “Methods” section. **a** HE staining; **b** IAR; **c** SP-A; **d** oxygenation index; **e** W/D; **f** PPI. Data were analyzed by one-way ANOVA and unpaired-samples T test. n = 10 for each group *P < 0.05 for comparisons of IR-C, IR-NS and IR-RV groups with Sham group; ^#^P < 0.05 for comparisons of IR-NS and IR-RV groups with IR-C group

The pathological changes of the four groups and the IAR results were demonstrated in Fig. 2a, b. The sham group showed no abnormal change; however, the lung tissue in IR-C and IR-NS group exhibited the damaged alveoli and inflammatory cells infiltration and other structure changes. In the group of RvD1 treatment, the tissue damages were reduced. For the IAR, all the three IR groups exhibited higher values than the sham group, which corresponded to the HE staining results (F = 297.43; P < 0.001). No difference of the IAR was found between the IR-C and IR-NS groups; however, compared to the IR-C group, the IAR in IR-RV was significantly higher (F = 162.62; IR-NS vs IR-C, P = 0.390; IR-RV vs IR-C, P < 0.001).

The results of SP-A, oxygenation index, lung tissue W/D and PPI were shown in Fig. 2c–f. The highest values of SP-A and oxygenation index were found in the sham group (F = 70.08; P < 0.01 and F = 48.40; P < 0.001 for SP-A and oxygenation index, respectively). After IR injury, both of them were decreased. Interestingly, the use of RvD1 brought the values back, which resulted in the statistic higher level in the IR-RV group when compared with the IR-C group (F = 65.68 for SP-A; IR-NS vs IR-C, P = 0.881; IR-RV vs IR-C, P < 0.001. F = 15.32 for oxygenation index; IR-NS vs IR-C, P = 0.152; IR-RV vs IR-C, P < 0.001). The W/D and PPI were increased in the IR groups when compared with the sham group (F = 87.81 and F = 505.43 for W/D and PPI, respectively; both P < 0.001); however, the IR-RV group showed significantly lower values of W/D and PPI compared with these in the IR-C group (F = 24.79 for W/D; IR-NS vs IR-C, P = 0.973; IR-RV vs IR-C, P < 0.001. F = 358.23 for PPI; IR-NS vs IR-C, P = 0.702; IR-RV vs IR-C, P < 0.001).

### The effects of RvD1 on IR-induced energy metabolism in lung tissues

**Fig. 3:**
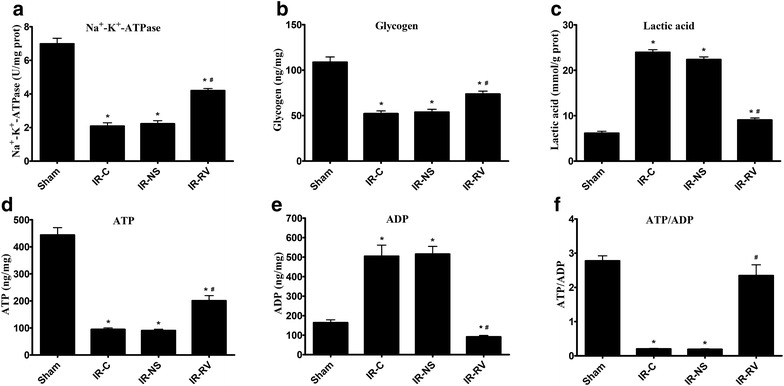
The effects of RvD1 on IR-induced energy metabolism in lung tissues. At T_2_, lung tissue was collected immediately after the IR procedure was completed and kept frozen in liquid nitrogen. Na^+^–K^+^-ATPase activity, glycogen, lactic acid, and ATP, ADP were measured as described in “Methods” section. **a** Na^+^–K^+^-ATPase; **b** glycogen; **c** lactic acid; **d** ATP; **e** ADP; **f** ATP/ADP. Data were analyzed by one-way ANOVA and unpaired-samples T test. n = 10 for each group *P < 0.05 for comparisons of IR-C, IR-NS and IR-RV groups with Sham group; ^#^P < 0.05 for comparisons of IR-NS and IR-RV groups with IR-C group

The results of Na^+^–K^+^-ATPase activity, glycogen and lactic acid levels in the lung tissues were shown in Fig. 3a–c. Compared to the sham group, the Na^+^–K^+^-ATPase activity and glycogen levels were remarkably decreased (F = 106.97 and F = 42.70 for Na^+^–K^+^-ATPase and glycogen, respectively; both P < 0.001). However, in the IR-RV group, the situation was better than the IR-C and IR-NS group, which showed significantly higher levels than the IR-C group (F = 47.26 for Na^+^–K^+^-ATPase; IR-NS vs IR-C, P = 0.584; IR-RV vs IR-C, P < 0.001. F = 13.49 for glycogen; IR-NS vs IR-C, P = 0.568; IR-RV vs IR-C, P < 0.001). The lactic acid level showed the opposite trend, which was increased in all the three IR groups (F = 271.65; all P < 0.01) but the lactic acid level in IR-RV was evidently lower than the IR-C group (F = 204.16; IR-NS vs IR-C, P = 0.069; IR-RV vs IR-C, P < 0.001).

The ATP, ADP and ATP/ADP in lung tissues were shown in Fig. 3d–f. At T_2_, when compared to sham group, the ATP and ATP/ADP levels were significantly decreased in IR-C and IR-NS group but with higher level of ADP (F = 95.48 and F = 38.78 and F = 61.85 for ATP, ADP and ATP/ADP, respectively; all P < 0.01). In IR-RV group, the ATP and ADP showed lower levels than the sham group (F = 95.48 for ATP; P < 0.001. F = 38.78 for ADP; P = 0.04). Compared to IR-C group, the ATP and ATP/ADP levels in IR-RV group were significantly higher but with lower ADP level (F = 28.83 for ATP; IR-NS vs IR-C, P = 0.928; IR-RV vs IR-C, P = 0.001. F = 35.73 for ADP; IR-NS vs IR-C, P = 0.998; IR-RV vs IR-C, P < 0.001. F = 45.88 for ATP/ADP; IR-NS vs IR-C, P = 0.895; IR-RV vs IR-C, P < 0.001). No changes were found in the levels of ATP, ADP and ATP/ADP between the IR-C and IR-NS group.

### The effects of RvD1 on inflammatory and oxidative stress reaction after LIRI

**Fig. 4:**
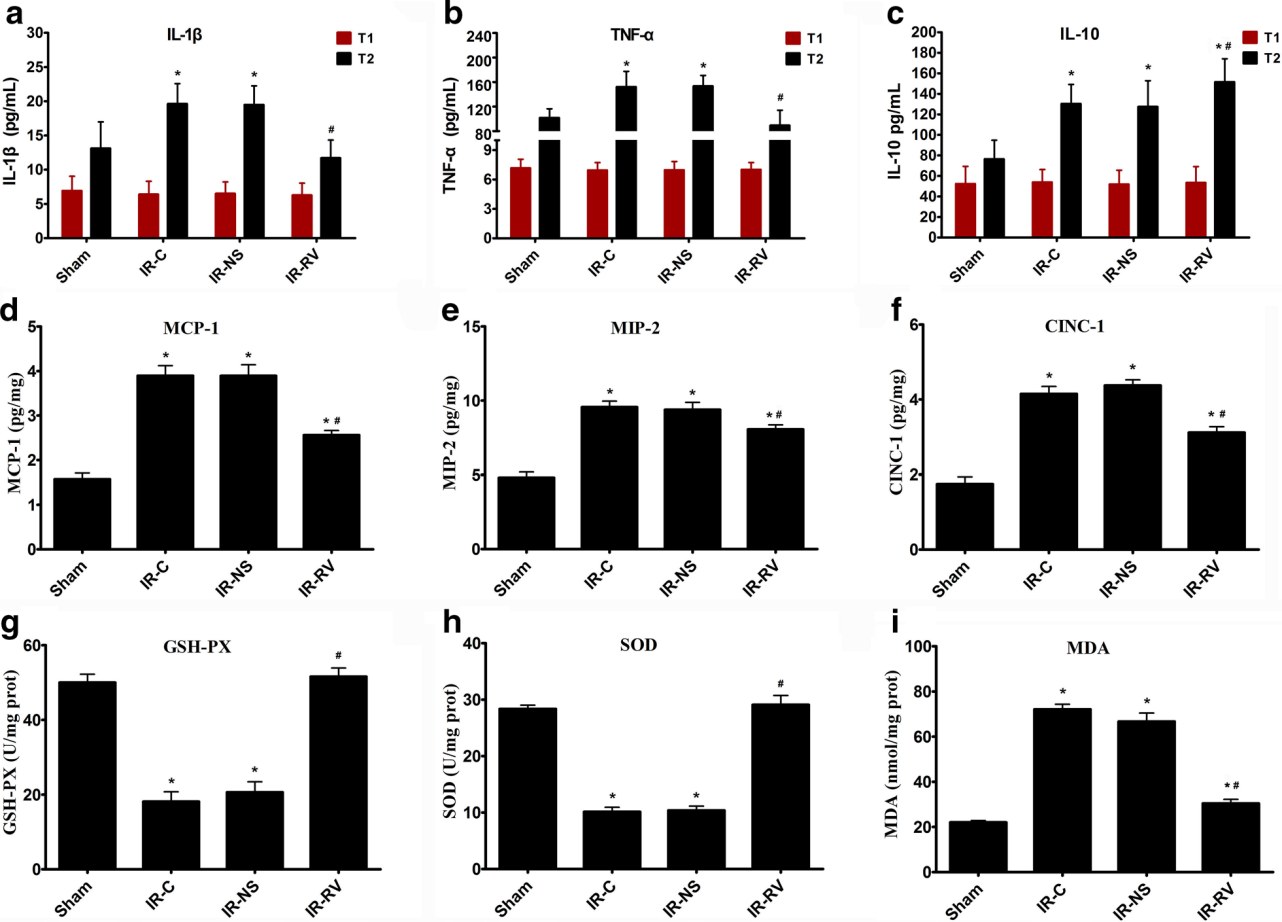
The effects of RvD1 on inflammatory and oxidative stress reaction. At T_1_, blood was collected immediately before thoracotomy. At T_2_, blood was collected after IR procedure was finished. At T_2_, lung tissue was collected immediately after the IR procedure was completed and kept frozen in liquid nitrogen. Inflammatory factors and GSH-PX, SOD, MDA were measured as described in “Methods” section. **a** IL-1β; **b** TNF-α; **c** IL-10; **d** MCP-1; **e** MIP-2; **f** CINC-1; **g** GSH-PX; **h** SOD; **i** MDA. Data were analyzed by one-way ANOVA and unpaired-samples T test. n = 10 for each group *P < 0.05 for comparisons of IR-C, IR-NS and IR-RV groups with Sham group; ^#^P < 0.05 for comparisons of IR-NS and IR-RV groups with IR-C group

The serum levels of IL-1β, TNF-α and IL-10 in the four groups were described in Fig. 4a–c. At T_1_, no difference was found among the four groups. At T_2_, the levels of IL-1β and TNF-α in IR-C and IR-NS group were higher than the sham group (F = 18.03 and F = 24.91 for IL-1β, TNF-α, respectively; both P < 0.001). However, there was no difference between the IR-RV and sham group (F = 18.03 for IL-1β; P = 0.316. F = 24.91 for TNF-α; P = 0.196). For the IL-10, all the three IR groups had higher levels than the sham group (F = 21.95; all P < 0.001). Compared to the IR-C group, the IL-1β and TNF-α levels in IR-RV group were significantly decreased but with increased level of IL-10 (F = 26.31 for IL-1β; IR-NS vs IR-C, P = 0.912; IR-RV vs IR-C, P < 0.001. F = 25.57 for TNF-α; IR-NS vs IR-C, P = 0.900; IR-RV vs IR-C, P < 0.001. F = 3.41 for IL-10; IR-NS vs IR-C, P = 0.776; IR-RV vs IR-C, P = 0.045). However, the three parameters showed no difference between the IR-NS and IR-C group.

The MCP-1, MIP-2, and CINC-1 content in lung tissues in each group were listed in Fig. 4d–f. At the time point T2, MCP-1, MIP-2 and CINC-1, levels in all the IR groups were higher than the sham group (F = 35.68, F = 33.13 and F = 45.51 for MCP-1, MIP-2, CINC-1, respectively; all P < 0.01). Compared to IR-C group, the levels of MCP-1, MIP-2 and CINC-1 decreased in IR-RV group (F = 14.28 for MCP-1; IR-NS vs IR-C, P = 1.0; IR-RV vs IR-C, P < 0.001. F = 8.58 for MIP-2; IR-NS vs IR-C, P = 0.99; IR-RV vs IR-C, P = 0.002. F = 15.32 for CINC-1; IR-NS vs IR-C, P = 0.359; IR-RV vs IR-C, P < 0.001). However, no changes were found between the IR-C and IR-NS group.

The GSH-PX, SOD activity and MDA content of the lung tissue in the four groups were shown in Fig. 4g–i. We could see that the GSH-PX and SOD activity in IR-C and IR-NS were significantly lower than the sham group (F = 52.51 and F = 103.11 for GSH-PX and SOD, respectively; both P < 0.001). No difference was found between the IR-RV and sham group; however, IR-RV group showed remarkably higher activity than the IR-C group (F = 51.81 for GSH-PX; IR-NS vs IR-C, P = 0.503; IR-RV vs IR-C, P < 0.001. F = 89.17 for SOD; IR-NS vs IR-C, P = 0.995; IR-RV vs IR-C, P < 0.001). As for the MDA production, all the IR groups showed higher value than the sham group (F = 120.34; IR-C vs sham and IR-NS vs sham, both P < 0.001; IR-RV vs sham, P = 0.049). However, after the using of RvD1, MDA production was significantly reduced in IR-RV group (F = 81.51; IR-NS vs IR-C, P = 0.170; IR-RV vs IR-C, P < 0.001).

### Effect of RvD1 on LIRI-induced cell apoptosis

**Fig. 5:**
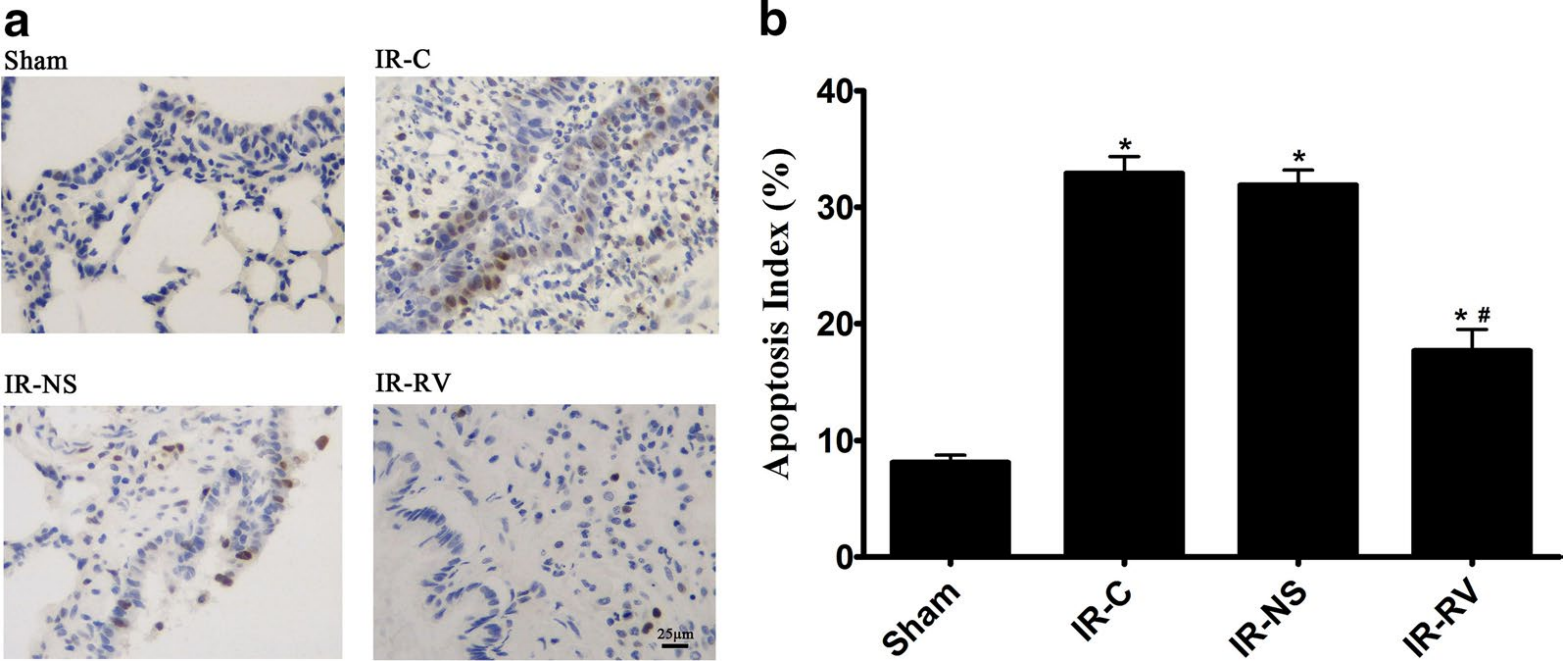
Inhibitory effect of RvD1 on LIRI-induced cell apoptosis. At T_2_, Apoptosis was determined by TUNEL assay according to manufacturer’s instructions. Cells with apoptotic morphological features and with tan or brown nuclei were judged to be apoptotic cells. **a** TUNEL assay, group Sham: a small amount of apoptotic cells in lung tissue; group IR-C and group IR-NS: apoptotic cells in lung tissue increased significantly; group IR-RV: apoptotic cells were between group Sham and group IR-C. **b** AI (apoptotic nuclei count/total nucleus count) was represent with histogram. Data were analyzed by one-way ANOVA and unpaired-samples T test. n = 10 for each group *P < 0.05 for comparisons of IR-C, IR-NS and IR-RV groups with Sham group; ^#^P < 0.05 for comparisons of IR-NS and IR-RV groups with IR-C group

The effect of RvD1 on lung tissue cell apoptosis at T_2_ was shown in Fig. 5a. Cells with apoptotic morphological features and with tan or brown nuclei were judged to be apoptotic cells. In Fig. 5a, only several apoptotic cells existed in the sham group, however, the number of apoptotic cells increased significantly in the IR-C and IR-NS group. Interestingly, after the treatment with RvD1, the apoptotic cells were decreased in the IR-RV group.

In terms of the AI (Fig. 5b), the three IR groups showed much higher values than the sham group (F = 77.05; all P < 0.001). No difference was found between the IR-C and IR-NS group. However, compared to the IR-C group, the AI in IR-RV was significantly reduced (F = 31.08; IR-NS vs IR-C, P = 0.647; IR-RV vs IR-C, P < 0.001).

## Discussion

LIRI is a pathological phenomenon, which refers to the rapid exacerbation of lung injury when lung tissue suffers from ischemia for a period and then blood supply is restored. Since LIRI happens more and more frequently in clinical practice in recent years, it is necessary to investigate its related mechanisms and the preventive or treatment methods [1, 23, 24]. Yet, the pathogenesis of the LIRI has not been fully clarified. Although many drugs were reported that they can reduce the LIRI in animal experiments, it is still very difficult to apply them into clinic due to their unsatisfactory effects or side effects [25–28]. Therefore, finding the safe and effective medications will provide great medical applications for the treatment of LIRI. The research about the mechanisms of LIRI has been focused on the following aspects: oxygen radicals, lipid peroxidation and excessive inflammatory response caused by neutrophil infiltration and inflammatory mediators [29]; however, there is very little research to study the energy metabolism disorder and its prevention, which was considered to be one of the causes of the LIRI and an important link in the pathogenesis of many other diseases [8, 30, 31].

Since the discovery of lipoxins, Rv has become a new lipid molecule that can reduce the inflammation and protect the tissue structure by restraining the proliferation of neutrophils, macrophages and other inflammatory cells [32–35]. The research about the protective effect of Rv on the IRI organs has just started and only few reports about the heart, brain and intestines can be found [14, 15, 36]. However, the effects of RvD1 on LIRI are still unknown.

The pathological changes of the lung tissue in the rat model of LIRI, especially the ultra-structural changes, can reflect the degree of lung injury in a more subtle way. Through TEM observation, we found that severe damage on the capillary alveolar respiratory membrane was caused by LIRI and other pathological changes such as the swollen type I epithelial cells, disappeared lamellar body, swollen mitochondria and dilated endoplasmic reticulum. HE staining showed the damaged alveoli, angiotelectasis, thickened alveolar septal infiltrated with inflammatory cells and the increased IAR level. With other data including the abnormality of W/D and PPI which reflected pulmonary edema and permeability as well as the AI in the three IR group, we confirmed that LIRI can cause severe lung injury. Fehrenbach et al. [37] thought the declining levels of SP-A after lung transplantation could indicate the progression of IRI. The preservation-dependent improvement of alveolar surfactant integrity after IR was associated with alterations in intra-alveolar SP-A levels. In lung transplantation experiments, the level of SP-A, SP-B and SP-C, markers of lung injury, in transplanted lungs treated with nitric oxide (NO) was decreased. Valiño et al. [38] concluded that the treatment with inhaled NO is deleterious for the surfactant system and causes a parallel worsening of arterial oxygenation. Therefore, SP-A level can be one of the indicators to LIRI. In this study, when the rats suffered from LIRI, the SP-A level and oxygenation index were significantly decreased, indicating the damage of the lung function. Interesting, we found that RvD1 could reduce cellular apoptosis in lung tissues, which is consistent with previous reports [39, 40]. The direct effects of RvD1 on the apoptosis of isolated lung cells should be tested in future studies. With the use of the RvD1, the AI was reduced significantly in IR-RV group, and the damage on the lung tissue was relieved with less pathological changes of the lung, less infiltrated inflammatory cells, lower W/D and PPI, and higher levels of SP-A and oxygenation index, suggesting the protective effect of RvD1 on the lung tissue when suffering from LIRI.

Some researchers believed that the energy metabolism disorder was the initiation of IRI [7]. Energy metabolism disorder, especially the deficiency of high energy phosphate compounds and carbohydrate is considered to be one of the main reasons of IRI [31, 41]. Extracorporeal circulation can cause damage to the internal organs, such as brain, kidney, lung, and liver. Dhein et al. [42] revealed that extracorporeal circulation decreased the ATP content in kidney and liver, but minocycline could significantly increase the ATP level in organs and reduce the injuries of kidney and liver. The Na^+^–K^+^-ATPase, a kind of protease, is located in the membranes of the cells and organelles functioning in substance transport, energy conversion, information transmission, cell membrane integrity maintenance and tissue metabolism. It can be a reliable index of the metabolic disturbance and tissue damage. Belliard [43] found that the Na^+^–K^+^-ATPase level on the cell membrane played a vital role in cell survival when studying the sodium pump and cell survival condition through an ischemia reperfusion model. The continuous intermittent low pressure hypoxia can relieve the reperfusion injury by enhancing the activity of Na^+^–K^+^-ATPase in guinea pig myocardium [44]. Due to the lack of substance, the energy synthesis by the mitochondria encountered obstacle, resulting in the decreased activity of the energy dependent Na^+^–K^+^-ATPase and finally a series of pathological response to the injury. Some degree of functional coupling of glycolytic ATP and Na^+^–K^+^-ATPase activity exists [45]. The decreased level of ATP and increased consumption of ATP should limit the Na^+^–K^+^-ATPase activity and activate the exchange of Na^+^–Ca^2+^, resulting in the overload of Ca^2+^. Moreover, the decrease of ATP content can also affect the activity of the calcium pump on cell membrane and in the cytoplasm, aggravating the overload situation of Ca^2+^. The increase of intracellular calcium increased the consumption of ATP; at the same time, Ca^2+^ overload can cause the accumulation of Ca^2+^ in the mitochondria, affecting the coupling of the electron transfer during the process of oxidative acidification and further aggravating the energy metabolic disturbance [46]. When suffered from IRI, Na^+^–K^+^-ATPase activity was inhibited, which would cause the cell toxicity, mitochondria and lysosomal membrane rupture, and finally increase inflammatory reaction and tissue damage [47]. On the other hand, Ca^2+^ overloading can increase the activity of many enzymes, and destroy the cell membrane, increasing the generation of free radicals and more damage to the lung tissue [48].

The results of this experiment showed that the decreased ATP level, the increased ADP level as well as the decreased ATP/ADP of the lung tissue appeared when rats suffered from LIRI. The content of ATP can not only reflect the process of oxidative respiratory chain of mitochondria and the ability of synthesizing high energy phosphates, but also the energy storage of the cells. The ratio of ATP to ADP (ATP/ADP) is a main factor that affects the oxidative phosphorylation. Decreased ratio refers to the weakened oxidative phosphorylation function and less ATP synthesis, which explained the increase of ADP content that was expected to be the result of the disturbance of synthesis of ATP. In addition, lower levels of glycogen and Na^+^–K^+^-ATPase activity as well as the higher lactic acid content caused by the acidism and glycolysis in the lung tissue confirmed that LIRI would cause evident energy metabolic disturbance. RvD1 can protect the structure and function of mitochondria and can further maintain the function of oxidative phosphorylation, promote ATP synthesis and delay the depletion of ATP, and finally recover the ratio of ATP to ADP. Meanwhile, it can enhance the activity of Na^+^–K^+^-ATPase to balance the homeostasis of the cells. Additionally, through up-regulating the glycogen content and down-regulating the lactic acid level, the energy metabolism was improved remarkably by RvD1, resulting in the less damage of the lung tissue when experiencing LIRI. Sukoyan et al. [49] agreed that the LIRI can be alleviated through improving cellular energy metabolism, such as protecting the mitochondrial oxidative respiratory activity, enhancing the ability of mitochondrial oxidative phosphorylation function and generation of high-energy phosphate compounds, as well as making the cells utilize the high-energy phosphate compounds. Ying et al. [50] thought that mitochondrial dysfunction was an important cause of IRI and the key factor to initiate apoptosis. This experiment is also observed RvD1 can reduce IR-induced apoptosis by improving energy metabolism and mitochondrial function,

There is still no clear evidence on whether RvD1 functions in the part of the energy metabolism, in a direct or indirect way. The relationship among the energy metabolic disturbance, inflammatory response and the oxidative stress response should be mutual connected and influenced as well as reciprocal causation. LIRI, characterized by inflammatory reaction and oxidative stress, can destroy the balance of the oxidative/anti-oxidative and pro-/anti-inflammatory systems. Many studies confirmed that the secretion of the inflammatory cytokines TNF-α, MIP-2, MIP-1, MCP-1 and CINC-1 would be increased during LIRI, which benefited the recruiting of neutrophils and further promote lung injury [51, 52]. In our study, we got the similar results. In particular, LIRI led to the large increase of the serum levels of IL-1, TNF-α, IL-10 and the concentrations of MCP-1, MIP-2 and CINC-1 in the lung tissue. In addition, more numbers of neutrophils and aggressive lung damage were observed in the lung tissue. Inflammation is associated with an oxidative stress reaction, which is produced in the development of inflammation, that which has positive feedback on inflammation itself [53]. Oxidative stress is considered to be an important pathogenic event in IRI. The deleterious effects of LIRI are in part mediated by the formation of free radicals and super oxides [54]. When the body suffered from ischemia, the function of the oxygen free radical scavenging system would be reduced or even totally loss; however, the activity of oxygen free radical generation system in the other way is enhanced. Once the blood supply is recovered, the oxygen free radicals would accumulate rapidly and further cause the damage of the tissue [55]. The scavenging of the free radicals is mainly achieved by a series of antioxidant enzymes, such as SOD and GSH-PX. Our experimental results showed that LIRI cause the imbalance between the production and elimination of the free radical, the increase of the MDA content, and the reduced activity of the SOD and GSH-PX [56, 57]. All the above results suggested that oxidative stress happened and the scavenging ability of free radicals was reduced, leading to the lung injury. At the same time, the increase number of white blood cell infiltration in the lung tissue can also produce a variety of free radicals and activate the lipid peroxidation of the cell membrane and damage the important components of the cells. Mitochondrial dysfunction and ATP level in the lung tissue are associated with various forms of lung injury and disease [58]. Study showed that due to the production of a large amount of free radicals, mitochondria experienced stress reaction, resulting in the decreased mitochondrial function, cellular energy metabolism disturbance, the reduced intracellular ATP, and low activity of Na^+^–K^+^-ATPase [59, 60]. In the present study, we found that LIRI could produce many kinds of toxic mediators, including oxygen free radicals, TNF, IL, and chemokines, which could recruit the neutrophil cells and other inflammatory cells, destroy the mitochondria structure and decrease the ATP level and the activity of the Na^+^–K^+^-ATPase, leading to the disturbance of the energy metabolism and further aggravate the tissue damage. RvD1 is able to restrain the infiltration of the inflammatory cells and the secretion of IL-1, TNF-α, MCP-1, MIP-2, and CINC, up-regulate the IL-10 level, enhance the SOD and GSH-PX activity and reduce the MDA content to restore the balance of the oxidant/antioxidant and pro-/anti-inflammatory system, and protect mitochondria. Therefore, the energy metabolism can be improved, leading to the less damage of the lung tissue.

RvD1 mainly functions in the inflammatory process, but it would not participate in the maintenance of the physiological functions. Therefore, this new type of anti-inflammatory drug will not interfere with the normal physiological activity and should cause no obvious adverse reaction in vivo. It is thus different from other anti-inflammatory agents, such as the glucocorticoid, the non steroidal anti-inflammatory drugs or the immune-suppressor, which usually induce a variety of adverse reactions. However, the unstable property, short half-life and the high price, the unknown dosage and the administrating timing, the uncertain frequency and delivery ways might limit its application. Therefore, the development of stable analogue could help to achieve the purpose of clinical application. In addition, the reported models and our animal models of LIRI were achieved by blocking and then loosing the pulmonary hilus, which not only blocked the pulmonary artery but also the bronchus and bronchial artery, leading to the difference from the clinical LIRI. The lung tissue has a dual blood supply system (pulmonary artery and bronchial artery) and these two systems anastomose extensively. Besides, the lung tissue can directly obtain oxygen by pulmonary ventilation, which makes the LIRI different from other organ’s IRI [61]. As a result, there may be some difference between this animal model of LIRI and the clinical situation. Much effort is still needed to improve the animal model of LIRI and make it closer to the clinical practice.

## Conclusions

In conclusion, RvD1 is able to improve the energy metabolism after LIRI, protect the mitochondrial structure and function from damage, increase the lung tissue ATP and glycogen content, recover the ATP/ADP ratio, enhance the Na^+^–K^+^-ATPase activity and decrease the lactic acid content. All the above effects may combine together, reduce the apoptosis in the lung tissue and protect the rats from LIRI. RvD1 can restore the balance of the oxidant/antioxidant and pro-/anti-inflammatory system. Therefore, the energy metabolism can be improved, leading to the less damage of the lung structure and function.

## Additional file

10.1186/s12967-016-0835-7 Product information.

## Abbreviations

LIRI: lung ischemia–reperfusion injury
IRI: ischemia–reperfusion injury
IR: ischemia–reperfusion
Rv: resolvin
RvD1: resolvin D1
SD: Sprague–Dawley
IR-C: ischemia–reperfusion-control
IR-NS: ischemia–reperfusion-normal saline
IR-RV: ischemia–reperfusion-resolvin
PBS: phosphate buffer solution
BALF: bronchoalveolar lavage fluid
HE: hematoxylin–eosin
TEM: transmission electron microscopy
IAR: injured alveoli rate
SP-A: pulmonary surfactant associated protein-A
W: wet weight
D: dry weight
PPI: pulmonary permeability index
IL: interleukin
TNF: tumor necrosis factor
MCP: monocyte chemoattractant protein
MIP: macrophage inflammatory protein
CINC: cytokine-induced neutrophil chemoattractant
GSH-PX: glutathione peroxidase
SOD: superoxide dismutase
MDA: malondialdehyde
AI: apoptosis index
NO: nitric oxide
TUNEL: TdT-mediated dUTP nick end labeling
HPLC: high performance liquid chromatography
