# Supplementary material for: Resolvin D1 mitigates energy metabolism disorder after ischemia–reperfusion of the rat lung
Source: J Transl Med. 2016 Mar 24;14:81. doi: 10.1186/s12967-016-0835-7 (PMC4806414; doi:10.1186/s12967-016-0835-7)
Supplement: Supplementary file 1 — 10.1186/s12967-016-0835-7 Product information. [file 12967_2016_835_MOESM1_ESM.pdf]

Product Information

Resolvin D1

Item No. 10012554

CAS Registry No.: 872993-05-0
Formal Name: 7S,8R,17S-trihydroxy-4Z,9E,11E,13Z,15E,19Z-docosahexaenoic acid
Synonyms: 17(S)-Resolvin D1, RvD1
MF: $C_{22}H_{32}O_5$
FW: 376.5
Purity: ≥95%
Stability: ≥1 year at -80°C
Supplied as: A solution in ethanol
Special Conditions: Light sensitive
UV/Vis.: λ_{max}: 302 nm

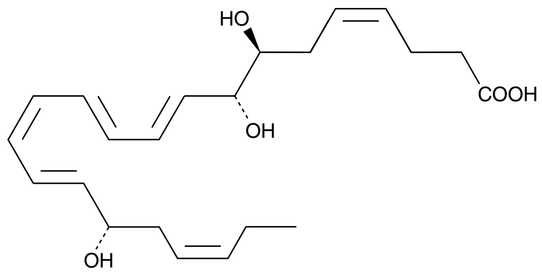

Laboratory Procedures

For long term storage, we suggest that resolvin D1 (RvD1) be stored as supplied at -80°C. It should be stable for at least one year.

RvD1 is supplied as a solution in ethanol. To change the solvent, simply evaporate the ethanol under a gentle stream of nitrogen and immediately add the solvent of choice. It is recommended that this product be stored and handled in an ethanol solution. Lipoxins can isomerize and degrade when put into freeze thaw conditions and/or in solvents such as dimethyl formamide or DMSO. If diluted with an aqueous buffer, this product should be discarded immediately after use.

Further dilutions of the stock solution into aqueous buffers or isotonic saline should be made prior to performing biological experiments. Ensure that the residual amount of organic solvent is insignificant, since organic solvents may have physiological effects at low concentrations. If an organic solvent-free solution of RvD1 is needed, it can be prepared by evaporating the ethanol and directly dissolving the neat oil in aqueous buffers. The solubility of RvD1 in PBS, pH 7.2, is approximately 0.05 mg/ml. We do not recommend storing the aqueous solution for more than one day.

Resolvins are a family of potent lipid mediators derived from both eicosapentaenoic acid (EPA; Item No. 90110) and docosahexaenoic acid (DHA; Item No. 90310).[1] In addition to being anti-inflammatory, resolvins promote the resolution of the inflammatory response back to a non-inflamed state.[2] RvD1 is produced physiologically from the sequential oxygenation of DHA by 15- and 5-lipoxygenase.[1] A 17(R)-epimer of RvD1 can also be generated in aspirin-treated samples.[3] Both RvD1 and its 17(R) configuration reduce human polymorphonuclear leukocyte (PMNL) transendothelial migration, the earliest event in acute inflammation, with EC_{50} values of ~30 nM.[4] RvD1 and its aspirin-triggered form also exhibit a dose-dependent reduction in leukocyte infiltration in a mouse model of peritonitis with a maximal inhibition of ~35% at a 10-100 ng dose.[4]

References

1. Hong, S., Gronert, K., Devchand, P.R., *et al.* Novel docosatrienes and 17(S)-resolvins generated from docosahexaenoic acid in murine brain, human blood, and glial cells. Autacoids in anti-inflammation. *J. Biol. Chem.* **278(17)**, 14677-14687 (2003).
2. Ariel, A. and Serhan, C.N. Resolvins and protectins in the termination program of acute inflammation. *TRENDS in Immunology* **28(4)**, 176-183 (2007).
3. Serhan, C.N., Hong, S., Gronert, K., *et al.* Resolvins: A family of bioactive products of ω-3 fatty acid transformation circuits by aspirin treatment that counter proinflammation signals. *J. Exp. Med.* **196(8)**, 1025-1037 (2002).
4. Sun, Y.-P., Oh, S.F., Uddin, J., *et al.* Resolvin D1 and its aspirin-triggered 17R epimer stereochemical assignments, anti-inflammatory properties, and enzymatic inactivation. *J. Biol. Chem.* **282(13)**, 9323-9334 (2007).

Related Products

For a list of related products please visit: www.caymanchem.com/catalog/10012554

WARNING: THIS PRODUCT IS FOR LABORATORY RESEARCH ONLY: NOT FOR ADMINISTRATION TO HUMANS. NOT FOR HUMAN OR VETERINARY DIAGNOSTIC OR THERAPEUTIC USE.

SAFETY DATA

This material should be considered hazardous until information to the contrary becomes available. Do not ingest, swallow, or inhale. Do not get in eyes, on skin, or on clothing. Wash thoroughly after handling. This information contains some, but not all, of the information required for the safe and proper use of this material. Before use, the user must review the complete Safety Data Sheet, which has been sent *via* email to your institution.

WARRANTY AND LIMITATION OF REMEDY

Cayman Chemical Company makes **no warranty or guarantee** of any kind, whether written or oral, expressed or implied, including without limitation, any warranty of fitness for a particular purpose, suitability and merchantability, which extends beyond the description of the chemicals hereof. Cayman **warrants only** to the original customer that the material will meet our specifications at the time of delivery.

Cayman will carry out its delivery obligations with due care and skill. Thus, in no event will Cayman have **any obligation or liability**, whether in tort (including negligence) or in contract, for any direct, indirect, incidental or consequential damages, even if Cayman is informed about their possible existence.

This limitation of liability does not apply in the case of intentional acts or negligence of Cayman, its directors or its employees.

Buyer's **exclusive remedy** and Cayman's sole liability hereunder shall be limited to a refund of the purchase price, or at Cayman's option, the replacement, at no cost to Buyer, of all material that does not meet our specifications.

Said refund or replacement is conditioned on Buyer giving written notice to Cayman within thirty (30) days after arrival of the material at its destination. Failure of Buyer to give said notice within thirty (30) days shall constitute a waiver by Buyer of all claims hereunder with respect to said material.

For further details, please refer to our Warranty and Limitation of Remedy located on our website and in our catalog.

Copyright Cayman Chemical Company, 06/11/2014

Cayman Chemical

Mailing address
1180 E. Ellsworth Road
Ann Arbor, MI
48108 USA

Phone
(800) 364-9897
(734) 971-3335

Fax
(734) 971-3640

E-Mail
custserv@caymanchem.com

Web
www.caymanchem.com
